# Identification of anti-tumour biologics using primary tumour models, 3-D phenotypic screening and image-based multi-parametric profiling

**DOI:** 10.1186/s12943-015-0415-0

**Published:** 2015-07-31

**Authors:** Alan M. Sandercock, Steven Rust, Sandrine Guillard, Kris F. Sachsenmeier, Nick Holoweckyj, Carl Hay, Matt Flynn, Qihui Huang, Kuan Yan, Bram Herpers, Leo S. Price, Jo Soden, Jim Freeth, Lutz Jermutus, Robert Hollingsworth, Ralph Minter

**Affiliations:** MedImmune, Granta Park, Cambridge, CB21 6GH UK; MedImmune, One MedImmune Way, Gaithersburg, MD 20287 USA; OcellO, Leiden BioPartner Center, J. H Oortweg 21, 2333 CH Leiden, The Netherlands; Retrogenix, Crown House, Bingswood Estate, Whaley Bridge, High Peak, SK23 7LY UK

**Keywords:** Non-small cell lung carcinoma, Phage display, Antibody, DARPin, 3-D phenotypic screening, Multi-parametric profiling, PDX, Cisplatin, CDCP1

## Abstract

**Background:**

Monolayer cultures of immortalised cell lines are a popular screening tool for novel anti-cancer therapeutics, but these methods can be a poor surrogate for disease states, and there is a need for drug screening platforms which are more predictive of clinical outcome. In this study, we describe a phenotypic antibody screen using three-dimensional cultures of primary cells, and image-based multi-parametric profiling in PC-3 cells, to identify anti-cancer biologics against new therapeutic targets.

**Methods:**

ScFv Antibodies and designed ankyrin repeat proteins (DARPins) were isolated using phage display selections against primary non-small cell lung carcinoma cells. The selected molecules were screened for anti-proliferative and pro-apoptotic activity against primary cells grown in three-dimensional culture, and in an ultra-high content screen on a 3-D cultured cell line using multi-parametric profiling to detect treatment-induced phenotypic changes. The targets of molecules of interest were identified using a cell-surface membrane protein array. An anti-CUB domain containing protein 1 (CDCP1) antibody was tested for tumour growth inhibition in a patient-derived xenograft model, generated from a stage-IV non-small cell lung carcinoma, with and without cisplatin.

**Results:**

Two primary non-small cell lung carcinoma cell models were established for antibody isolation and primary screening in anti-proliferative and apoptosis assays. These assays identified multiple antibodies demonstrating activity in specific culture formats. A subset of the DARPins was profiled in an ultra-high content multi-parametric screen, where 300 morphological features were measured per sample. Machine learning was used to select features to classify treatment responses, then antibodies were characterised based on the phenotypes that they induced. This method co-classified several DARPins that targeted CDCP1 into two sets with different phenotypes. Finally, an anti-CDCP1 antibody significantly enhanced the efficacy of cisplatin in a patient-derived NSCLC xenograft model.

**Conclusions:**

Phenotypic profiling using complex 3-D cell cultures steers hit selection towards more relevant in vivo phenotypes, and may shed light on subtle mechanistic variations in drug candidates, enabling data-driven decisions for oncology target validation. CDCP1 was identified as a potential target for cisplatin combination therapy.

**Electronic supplementary material:**

The online version of this article (doi:10.1186/s12943-015-0415-0) contains supplementary material, which is available to authorized users.

## Background

Antibody therapies that target tumour antigens are now well established in the arsenal of anti-cancer treatments. However, a major challenge in expanding the range of tumours treatable by this product class is the identification of new, antibody-tractable targets. Transcriptomics and proteomics can assist in identifying potential antigens, but these methods do not reveal whether an antibody-mediated therapy will have any impact on tumours. An alternative approach to finding novel targets is phenotypic antibody screening, where panels of antibodies selected against disease cell types are screened in a target-agnostic manner for a desired functional effect on tumour cells, prior to performing target identification. Similar approaches are well established for identifying small molecule therapeutics, where they are recognised in particular for their ability to find first-in-class therapies [1]. Antibody-based phenotypic screening has been described previously by ourselves [2], and others [3–5], but all reports to date have focussed on established tumour cell lines as a screening platform. Here we report a functional antibody screen using primary cells from non-small cell lung cancer (NSCLC) patients, grown in spheroids and in anchorage-independent culture conditions that aim to replicate more closely the phenotypes of tumours in patients.

Immortalised tumour cell lines grown in two-dimensional (2-D, monolayer) cultures are a popular platform for *in vitro *screening of novel anti-cancer therapeutics, due to their ease of culture, reproducibility and analysis, which all facilitate the performance of high-throughput discovery campaigns. However, these cells have intrinsic limitations for drug discovery, as their response to therapy often differs from disease tissue in patients, and hence 2-D cell-line based assays do not consistently predict efficacy of therapeutics in clinical trials [6]. To help avoid late-stage drug development failures, more relevant *in vitro *screens are being sought, using primary cells or co-cultures, grown in more complex culture formats, to model the disease mechanisms in real tissues more closely [7]. The choice of xenograft models used for assessing therapeutic efficacy *in vivo *has a similar bearing on disease relevance. Patient-derived xenograft (PDX) models, using primary tumours directly transferred from the patient into an immunodeficient mouse and maintained by passaging cells from mouse to mouse, can retain more closely the phenotype of real patient tumours when compared to cell line-derived xenografts, including gene expression profiles [8] and histology [9–11]. Even limited passage in tissue culture can be detrimental to xenografts models–a study of small cell lung cancer (SCLC) xenografts found that PDX models retained a tumour-specific gene expression signature also seen in primary SCLC tissue, which was irreversibly lost when the cells were transitioned to tissue culture and then re-established as secondary xenografts [6].

For high-throughput drug discovery programs, the cell culture models need to be compatible with the requirements of the screening platform. Complex 3-D culture methods are now well established for both normal cells [12–14] and tumour models [15], but it is still challenging to use them in large-scale screens, where reproducibility and the sensitivity of detection methods are essential to the success of a screening campaign. In this study, we investigated screening models developed from primary non-small cell lung carcinoma (NSCLC) cells from human donors, and characterised their suitability for screening biologics in different culture formats. Two models proved to be suitable for screening in spheroid cell cultures, anchorage-independent cultures, and in standard monolayer cultures. Spheroid cultures (reviewed by Fennema et al. [14]) use cells grown in small aggregates that are thought to allow more natural cell-cell and cell-matrix contacts to develop. The anchorage-independent cultures were used to test for antibodies interfering with anoikis resistance–the ability of cells to avoid apoptosis in the absence of normal cell-cell contacts, an important pre-requisite for metastasis [16]. We generated a panel of antibodies in two molecular formats by performing phage-display selections against the primary NSCLC cells; a designed ankyrin repeat protein (DARPin) antibody mimetic library was used, in addition to a scFv library. The selected molecules were then screened for anti-proliferative and pro-apoptotic effects in assays, without knowledge of their targets, using primary NSCLC tumour cells grown in spheroids, monolayers and in anchorage-independent culture. We believe this is the first report of a large-scale target-naïve functional screen for novel biologics performed using primary cells in complex assay formats.

In order to enrich the phenotypic information on the effects of these molecules, a subset of the DARPins was also profiled in an image-based high content screen using a tumour cell line cultured in a complex 3-D matrix. Multi-parametric phenotypic profiling [17–19] was applied to construct statistical models to discriminate the phenotypes induced by treatment, without prior selection of the measurement parameters. We compared treatment-induced effects of the DARPins on cell morphology and invasion phenotypes, and through this analysis identified distinct effects within a set of DARPins that bind to CUB-domain containing protein 1 (CDCP1). CDCP1 is a cell surface transmembrane protein that is widely expressed on many cell types, but also upregulated on many tumour cells and cell lines. Its function has been associated with invasive and metastatic phenotypes (reviewed by Uekita and Sakai [20]), including models of prostate cancer [21–23], and it was recently linked to Ras-driven invasiveness and upregulation of matrix degradation [24].

Finally, we tested an anti-CDCP1 IgG antibody derived from our panel in a NSCLC PDX model, both as a single agent and in combination with cisplatin treatment. We saw no efficacy from antibody treatment alone in inhibiting growth of this patient-derived tumour model, in contrast to similar studies performed using other CDCP1 antibodies in cell line xenograft models [25]. However, the antibody treatment led to significant enhancement of tumour growth inhibition when co-administered with cisplatin.

## Results

### Characterisation of primary NSCLC cells as *in vitro *and *in vivo *screening models

**Fig. 1:**
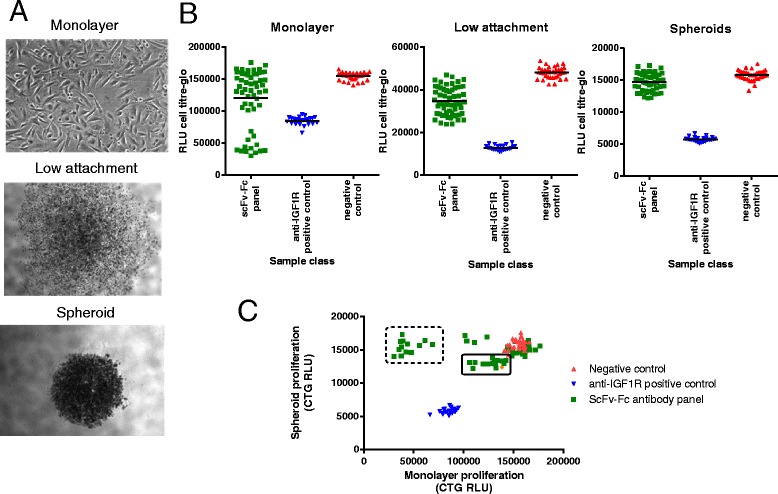
Phenotypic screening in primary NSCLC tumour cells. **a** Images of cells derived from NSCLC primary tumour #1 cultured in three different conditions. **b** Effects of the scFv-Fc antibody panel upon NSCLC tumour #1 cell growth in three culture conditions, measured by Cell-Titre Glo (CTG) luminescence signal. Each antibody was individually dosed (without normalising concentrations) and cells were grown in 96-well plates. Positive (anti-IGF1R) and negative control antibodies were dosed in multiple replicates on each plate to establish consistency between plates. Each data point indicates a single well. *Black horizontal bars* indicate the average value for a sample class. **c** Scatter plot comparing the effects of scFv-Fc antibodies on NSCLC tumour #1 cell growth grown in spheroids and in standard monolayer cultures. Each data point indicates a single antibody (or replicate of the controls). The *dashed box* indicates a group of antibodies that strongly inhibited growth of cell monolayers but not spheroids. The *solid*-*line box* indicates a group of antibodies with a weak inhibitory effect in both spheroids and monolayers. The *orange*-*coloured* datapoint represents an antibody that was later shown to bind CDCP1 (αCDCP1-Ab3 in Fig. 2)

Cells from three NSCLC tumours (see Table 1) were investigated to determine their suitability for *in vitro *screening assays. Of the three tumours, in vitro assays were feasible for tumours #1 and #2 in three culture modes: spheroid, low-attachment (anoikis), and monolayer (Fig. 1a and Additional file 1: Figure S1). Tumour 1 was most amenable to screening, giving reproducible and dose-dependent reductions in cell numbers in response to positive control anti-IGF1R antibody treatment in all three assay formats, measured by Cell-Titer Glo luminescence to detect total cellular ATP (Additional file 1: Figure S1A). The same treatment also induced caspase 3/7 activity in tumour #1 in spheroid and in low attachment cultures (Additional file 1: Figure S1B). Cells from tumour #2 were less sensitive to anti-IGF1R treatment in all assay formats, but responded to combination treatment with anti-IGF1R and anti-EGFR antibodies (Additional file 1: Figure S1C/D). Tumour #3 failed to grow well in tissue culture, but was suitable for propagation *in vivo *when passaged within immunodeficient mice, and was used to establish an NSCLC PDX model. Hence, cells from tumours #1 and #2 were selected for antibody isolation by phage display, and cells from tumour #1 were also used for *in vitro* functional screening.

**Table 1:** Characteristics of the NSCLC primary tumours tested

Tumour #	Gender, age at excision	Site	Clinical diagnosis of specimen	AJCC/UICC stage group	Medications	Genetic analysis
1	F, 79	Lung	Squamous cell carcinoma of the lung	IA		SNPs: rs2075607 (LKB1), rs1042522 (TP53)
2	M, 57	Lung	Adenocarcinoma of the lung	IIB	Combivent	nd
					Lovenox	
					Advair	
3	M, 61	Abdominal wall	Metastatic neoplasm of the abdominal wall	IV	Carboplatin	Highly aberrant copy number variations across genome
					Paclitaxel	Point mutations: KRas G12D, TP53 V157F
					Alimta	SNPs: rs2075607 (LKB1)
					Tarceva	
					Gemcitabine

### Phage display selections on primary NSCLC cells

Phage display with scFv and DARPin libraries was performed using a mixture of cells from NSCLC tumours #1 and #2 as the selection antigen. Up to three successive rounds of cell panning were performed to enrich for phage able to bind to the cells. The selected antibodies (encompassing both the scFv and DARPin molecular formats) were screened for binding to cells from NSCLC tumours #1 and #2, as well as to a panel of established cell lines, using crude extracts from *E. coli* expression. Seventy-eight (13 %) of the scFv antibodies bound to at least one cell type, as did 231 (22 %) of the DARPins; these cell-binding antibodies were converted to Fc-fusions, expressed in mammalian cell culture and purified for testing in phenotypic screens.

### Proliferation and apoptosis phenotypic screens

We performed a screen to test for functional effects of the panel of antibodies upon cells from tumour #1 cultured in the three different formats established above, measuring overall proliferation in all three culture conditions in the presence of antibodies, and induction of apoptosis in the spheroid-forming and low-attachment conditions. The choice of culture format clearly modulated the response of the cells to treatment with scFv antibodies (Fig. 1b and Additional file 2: Figure S2A); the cells grown as spheroids were in general less sensitive to the antibodies compared to those in low-attachment conditions. In monolayer cultures, a subset of the antibodies showed stronger anti-proliferative effects than observed with the anti-IGF1R positive control, behaviour which was not replicated in the spheroid cultures (Fig. 1c, dashed box). Instead, a different population was identified (Fig. 1c, solid box) that was moderately active in spheroid culture conditions. For the DARPin-Fc fusions, the overall sensitivity to treatment in the spheroids was higher than was observed with the scFv antibodies. A small number of DARPins showed pro-proliferative effects that were not seen in monolayer culture (Additional file 2: Figure S2B).

**Fig. 2:**
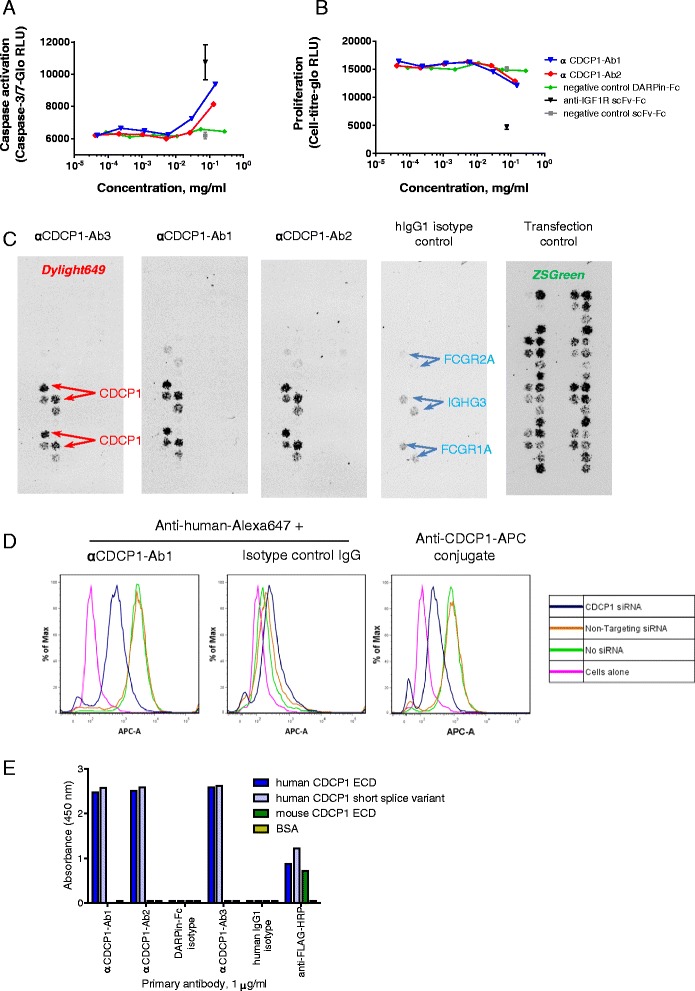
Identification of CDCP1-binding antibodies with activity against NSCLC tumour cells. **a** + **b** Dose-dependent activity of two antibodies that induced caspase 3/7 activation and inhibited proliferation in primary NSCLC tumour #1 cells grown as spheroids. **c** CDCP1 was identified as the antigen of the two antibodies shown in (a/b), and also of the antibody indicated in orange in Fig. 1c. Target ID was performed using cell-surface display of a human membrane protein cDNA array in HEK293 monolayers. Array positions were determined using zsGreen encoded within the cDNA library vector. Antibody binding was detected via a Dylight649-labelled secondary antibody. **d** siRNA knockdown of CDCP1 in NCI-H358 cells reduced the binding of αCDCP1-Ab1 (and αCDCP1-Ab2–see Additional file 3: Figure S3A), and of positive control anti-CDCP1 antibody (clone 309121-APC conjugate), as determined by flow cytometry. **e** The anti-CDCP1 antibodies identified in Fig. 2c all recognise the N-terminal region of CDCP1 that is shared by both splice variants, shown by direct ELISA on FLAG-His10-tagged recombinant antigens. The antibodies are not crossreactive to mouse CDCP1

Antibodies that showed signs of either anti-proliferative or pro-apoptotic effects on the primary NSCLC cells, in either the spheroid or low-attachment conditions, were studied further to look for dose-dependent effects on the cells. Two examples are shown in Fig. 2a and b, where a pair of antibodies, both subsequently identified as binding CDCP1, caused induction of caspase 3/7 activity and inhibited proliferation in NSCLC tumour #1 spheroids.

### CDCP1 is the target antigen of several functionally active antibodies

To identify the antigens recognised by antibodies that showed activity in functional screens, we used an arrayed cDNA library of human membrane proteins transfected into HEK293 cells [26]. The resulting array of cell surface-displayed membrane proteins was fixed and incubated with individual antibodies, followed by fluorescent detection. Among the identified antibody:antigen interactions, we found three antibodies (two DARPin-Fcs, designated αCDCP1-Ab1 and αCDCP1-Ab2, and one IgG, αCDCP1-Ab3) that specifically recognised cells transfected with CUB-domain containing protein 1 (CDCP1) (Fig. 2c). CDCP1 is a type 1 transmembrane protein with a large extracellular domain that is upregulated in many tumour types, and has been linked functionally to anoikis resistance, tumour invasion and metastasis [24, 27–30]. Binding to CDCP1 by our antibodies was confirmed by flow cytometry on NCI-H358 lung cancer cells, where siRNA knock-down of CDCP1 reduced the level of antibody binding (Fig. 2d and Additional file 3: Figure S3A), and by direct ELISA performed using recombinant full-length CDCP1 (Additional file 3: Figure S3B).

To further characterise binding to CDCP1, we generated three recombinant protein constructs; the extracellular domains (ECDs) of both human and mouse CDCP1, and a shorter splice variant of human CDCP1 (described as isoform 2 by Perry et al. [31] but as isoform 3 in the Uniprot database). The short isoform’s mRNA is expressed at similar levels to the long isoform in many cell types [31] and is of unknown function. Since it has a signal peptide but no transmembrane domain, it is potentially a secreted factor. All three proteins were expressed in HEK293 cells and purified by immobilised metal affinity chromatography (IMAC) followed by size exclusion chromatography (SEC). The recombinant CDCP1 fragments were used as antigens in direct ELISAs, which revealed several additional CDCP1-binding DARPin-Fcs in our panel that were not prioritised in the functional screens on NSCLC cells. All the anti-CDCP1 molecules identified by ELISA recognised both splice-forms of human CDCP1, indicating an epitope within the shared *N*-terminal region of the extracellular domain (Fig. 2e). None were cross-reactive to murine CDCP1. Also consistent with an epitope in the shared N-terminal region, neither αCDCP1-Ab1 nor αCDCP1-Ab3 inhibited cleavage of the full CDCP1 extracellular domain by recombinant matriptase catalytic domain at a proteolytically sensitive site, which is located outside the region shared by both isoforms (Additional file 4: Figure S4A). However, incubation of αCDCP1-Ab3 with three different cell lines in monolayer culture did reduce the level of proteolytically truncated CDCP1, as has been seen for other anti-CDCP1 antibodies [29, 32]; this effect appears to be due to antibody-mediated decreases in overall CDCP1 levels rather than protection from proteases (Additional file 4: Figure S4B).

### 3-D multi-parametric profiling

**Fig. 3:**
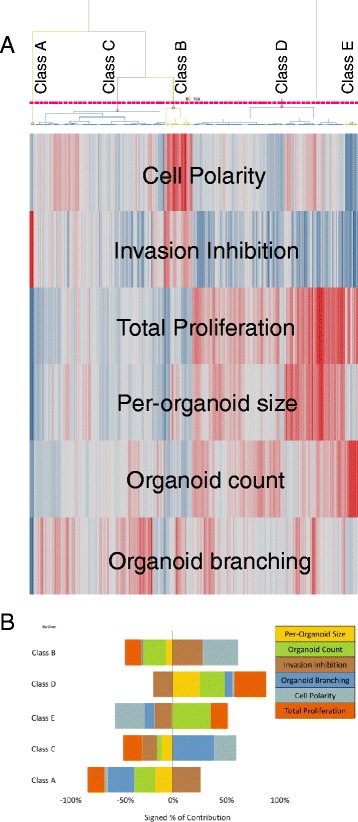
Antibody-induced phenotypes in 3-D cultured PC-3 cells in a high content screen. **a** Clustering of antibody-induced phenotypes in a high content screen performed using PC-3 cells grown in a 3D matrix. Each antibody was dosed at six different concentrations and in four replicates. Phenotypic measurements were determined from images and analysed as described in Methods. The replicates were averaged prior to clustering analysis, but different doses of each antibody were analysed separately. The dendrogram shows the clustering pattern of individual samples into 5 phenotypic classes, A–E (see also Additional file 5: Table S1. The heat map represents the values for six phenotypic features that distinguished these classes (*red* = positive; *blue* = negative)–see Methods for definitions of these features. **b** Bar chart showing the contributions of the six phenotypic features, either positively or negatively, to the five phenotypic classes used to assign antibody-induced effects on the PC-3 cells

To enable clearer discrimination between the antibodies based on sub-effective doses, and to enrich the phenotypic data beyond relatively simple measures such as proliferation, we screened a panel of the DARPin-Fc antibodies in a 3-D multi-parametric assay [33], using PC-3 prostate cancer cells grown in protein hydrogels in 384-well plates. These cells form a complex three dimensional phenotype that facilitates measurement of changes in morphological phenotypes in a high content screen. The cells were grown in the presence of DARPin-Fc antibodies at different concentrations. αCDCP1-Ab1 and dasatinib were included as positive controls, and non-binding antibodies as negative controls. A broad DARPin-Fc antibody panel was used, which was not chosen on the basis of functional effects observed in the primary NSCLC cells, since we wished to detect effects on the cells that were not captured by measurement of proliferation or caspase induction. All treatments were performed in quadruplicate. Following treatment, samples were fixed and stained to visualise filamentous actin and nuclei, then 3-D image stacks were collected and analysed, retaining spatial information in the z-plane, to generate a set of 294 different measured features. To identify a minimal set of robust features that could best classify the different treatments, machine learning and hierarchical clustering were applied to a pairwise comparison between control samples and each individual DARPin dose. This analysis resulted in an optimum set of six features (see Methods for definitions); Invasion Inhibition, Organoid Count, Total Proliferation, Cell Polarity, Organoid Branching and Per-Organoid Size. Scores for these features divided the antibody-induced phenotypes into 5 classes, designated A to E (see Fig. 3 and Additional file 5: Table S1).

Many of the DARPin-Fc antibodies had no effect on PC-3 growth or morphology, which may reflect either a lack of function and/or the absence of their antigens on these cells. For the antibodies that did induce changes in the cells, the phenotypes induced by individual antibodies at different concentrations frequently fell into different classes, confounding classification of antibodies to a specific phenotypic class. In some cases, this was because low doses had no effect on the cells, but in other cases (notably αCDCP1-Ab1), morphological effects observed at low doses were masked at higher concentrations by an overall reduction in cell numbers, emphasising that simple end-point read-outs such as proliferation do not capture the full effect of the antibodies on the cells. We therefore assigned separate phenotypic class labels to each dose tested, then antibodies were assigned a general phenotypic class based on the prevalence of a particular class across the dose range.

### Two categories of anti-CDCP1 DARPin-Fcs cause distinct functional effects in PC-3 cells

**Fig. 4:**
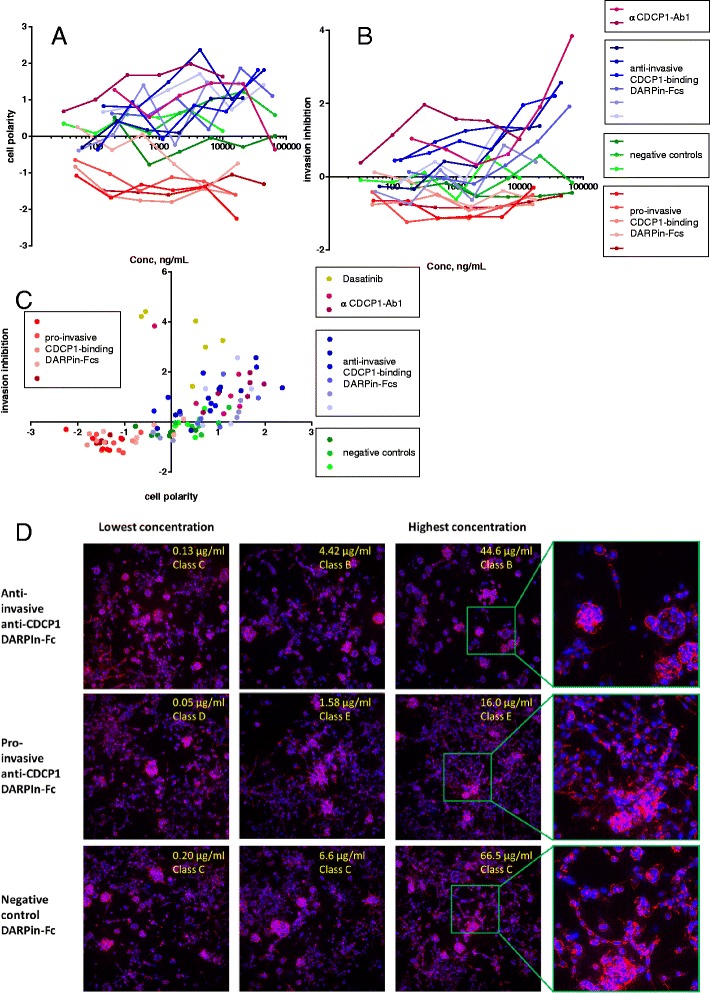
Opposing effects of two groups of anti-CDCP1 molecules upon PC-3 cell invasiveness. CDCP1-binding DARPin-Fcs segregate into two populations in the phenotypic space determined by the multiparametric high-content screen performed on PC-3 cells: plots showing the effects on cell polarity (**a**) and invasion inhibition (**b**) . Group 1 (*blue symbols*/*lines*) behave similarly to αCDCP1-Ab1 (in *purple*), inhibiting invasion and somewhat increasing cell polarity. Group 2 (in *reds*) decrease polarity, and increase invasiveness. Control antibodies are shown in green. **c** Scatter plot showing a correlation between cell polarity and invasion inhibition for the same dataset as in panels a/b. Each point represents a single concentration of an antibody, using the same colour scheme as in (a/b). **d** Example images from the high content screen, showing the morphological effects of different classes of anti-CDCP1 antibodies on PC-3 cells grown in 3D culture. Each row shows image stacks of cells cultured in the presence of an anti-invasive anti-CDCP1 (*top row*) pro-invasive anti-CDCP1 antibody (*middle row*) or negative control antibody (*bottom row*), at increasing concentration from left to right. The fourth column shows an enlarged view of the indicated areas. Staining: *red* = filamentous actin, *blue* = nuclei

The high content multi-parametric screen performed on PC-3 cells included several of the anti-CDCP1 DARPin-Fcs that were isolated by phage selections against the NSCLC tumour cells. Only a subset of these CDCP1-binders was active in the functional assays on NSCLC primary cells; others were found later by ELISA on recombinant protein after the target of the active antibodies had been identified. To understand why molecules with a common antigen did not also have a common functional effect on the cells, we examined in more detail the induced phenotypic effects of these molecules on PC-3 cells. Anti-CDCP1 molecules had some of the strongest effects in the multiparametric screen, but interestingly these molecules divided into two subsets. The first group (blue lines in Fig. 4a/b) induced a class B phenotype in PC-3 cells, which shows lower invasiveness and a more polarised organellar phenotype compared to cells treated with negative controls. αCDCP1-Ab1, which was identified as a hit in the functional screens in NSCLC cells, behaved similarly but was more potent and induced a class A phenotype due to reduced overall cell numbers. Within this group, well-defined structures were observed with polarised cells surrounding lumens (Fig. 4d, top row). (Interestingly, we also saw changes in cell growth morphology with αCDCP1-Ab3 treatment in low attachment conditions during the earlier dose–response functional screens in cell lines–see Additional file 6: Figure S5A). A very different effect was seen in the second group of anti-CDCP1 antibodies (red lines in Fig. 4a/b), which trended towards phenotypic class E, causing increased invasiveness and decreased cell polarity (Fig. 4d, middle row). In fact, across the whole dataset, only seven DARPin-Fcs induced a class E phenotype, five of which were from the CDCP1-binding group.

### In vivo anti-CDCP1 enhances the effect of cisplatin against a NSCLC PDX model

**Fig. 5:**
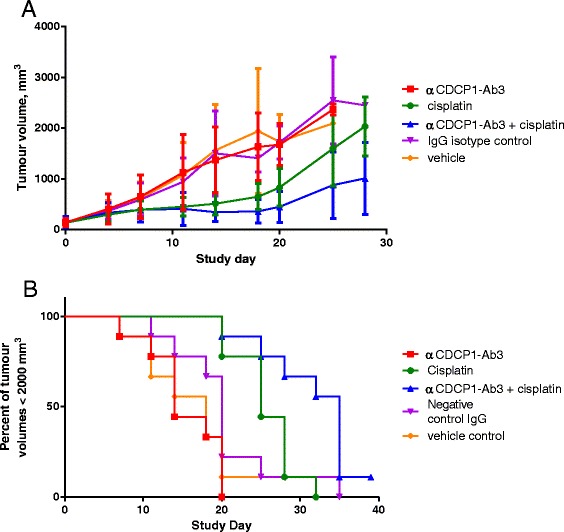
anti-CDCP1 therapy enhances cisplatin tumour growth inhibition in a patient-derived NSCLC xenograft model. αCDCP1-Ab3 delivered at 30 mg/kg causes enhanced tumour growth inhibition of a stage IV metastatic NSCLC tumour when co-administered with cisplatin, but has no effect as a single agent. **a** Tumour growth curves for patient-derived NSCLC xenografts, treated with anti-CDCP1, cisplatin or a combination. Data shown are averages ± SD for 9 mice per treatment arm. **b** Survival curves for reaching 2000 mm^3^ tumour volumes. The anti-CDCP1 + cisplatin combination significantly extended the time to reach 2000 mm^3^ tumours over cisplatin alone, with median survival of 33.5 v. 25.0 days, *p* = 0.011 (Mantel-Cox test)

Due to the strong functional and phenotypic effects seen in primary NSCLC tumour cells in 3-D screening assays treated with CDCP1-binding antibodies, the activity of αCDCP1-Ab3 was tested for tumour growth inhibition *in vivo *using patient-derived xenografts of Tumour #3, a stage IV recurrent, metastatic NSCLC tumour, implanted subcutaneously in immunodeficient mice. The mice were treated with the antibody at 30 mg/kg (i.v. 6 doses, twice per week for 3 weeks), in comparison with vehicle or a negative control antibody. Also included were cisplatin (6 mg/kg i.v. 3 doses, 4 days apart) and antibody/cisplatin combination dosing. Tumour volumes were measured twice weekly until they reached 2000 mm^3^. We observed no effect from anti-CDCP1 (αCDCP1-Ab3) treatment alone on tumour growth, compared to vehicle or a negative control antibody (Fig. 5a). However, the same antibody did cause a retardation of tumour growth when co-administered with cisplatin, relative to the retardation due to cisplatin alone at the same dose. Survival curves for growth to >2000 mm^3^ tumours were significantly longer for cisplatin + αCDCP1-Ab3 than for cisplatin alone, with median survival times of 33.5 v. 25.0 days (Fig. 5b).

## Discussion

In order to meet the significant unmet need for novel therapeutics in all disease areas, including oncology, there remains a desire to identify new, drug-tractable targets for both small molecule and biologic drugs. Biological therapeutic discovery is currently dominated by a target-based paradigm, often built on targets identified from expression data in disease models. In recent years, however, there has been a renaissance in the use of target-agnostic, phenotypic screening for small molecule drugs following analyses that showed this route is particularly effective at finding first-in-class molecules [1]. More subtle analyses have shown that therapeutics are often discovered by combining phenotypic and target-led screens, but that an initial phenotypic screen can be an effective way to identify initial leads [34]. Target-agnostic discovery is less common for biologics, but a growing number of reports have emerged [2–5]; the hope is that such screens can provide an alternative route to novel antibody therapeutics.

The therapeutic relevance of a functional screen obviously depends on the quality of the screen itself, which to be successful should mimic the disease state as closely as possible. This can require a level of complexity that can be difficult to incorporate into a robust and reproducible screen. In order to identify novel antibody-tractable therapeutic targets by phenotypic screening, we assessed three different patient-derived NSCLC tumours for their suitability for use as a screening platform. Two models were suitable for reproducible *in vitro *screening, while one model could only be maintained *in vivo*. Biologics were selected against two of the primary cell populations by phage-display, using both scFv and DARPin antibody libraries. DARPins were included in our selections, due to in-house data showing improved levels of phage display, making it easier to perform cell-surface selections where the availability of cells was limiting. The resulting molecules were screened against primary cells grown in three different culture formats–a standard monolayer, a 3D spheroid-forming culture, which allows more native-like contacts between cells, and a low-attachment “anoikis-promoting” culture that aims to force cells to rely on survival pathways that are important during metastasis. Our data showed that efficacy of biologics against cells in monolayers often did not correlate well with efficacy against the same cells grown in spheroid or low-attachment cultures. Had our *in vitro *screening only used monolayer-based assays, subsequent studies could have focussed on agents that only showed activity in the least disease-relevant culture format. Instead, by including screens on other culture formats, we hope to have identified hits with higher confidence that the mechanism of action will translate to the clinic.

After screening for antibodies with functional activity against primary NSCLC cells in the proliferation and apoptosis screens, we wished to identify the most promising candidates for more detailed study. Target identification was performed using a cell-surface array of membrane proteins, presented by HEK293 cells, which should help ensure antigens were correctly folded. This method led to the identification of CDCP1 as the antigen of several of our antibodies. However, it was clear from our dose–response data that the antibody treatments routinely did not achieve their maximal or EC50 effects on the measured functional endpoints over the concentrations tested (see, for example, Fig. 2a for two anti-CDCP1 DARPins). This may be due to relatively weak antigen-binding affinities for these antibodies derived from naïve phage-display libraries without affinity maturation. We were most interested in identifying hits with novel targets or mechanisms of action, not those with the highest affinity or lowest EC50 (which is better addressed during lead optimisation). We had also noticed that additional CDCP1-binding antibodies identified in the NSCLC-selected panel had no effect in the functional assays on primary cells, and we wished to understand why. Therefore, having identified a panel of antibodies showing either binding to, or activity against the primary NSCLC tumour material, we also used a multiparametric screen to determine the phenotypic effects of the antibodies in more detail. For this, we used small volume 3-D microtissue cultures of PC-3 cells, grown in protein hydrogels that allow the cells to form a complex, invasive architecture. Although this involves a switch to a prostate cell line in place of lung cancer primary cells, which may be disadvantageous in translating the molecular effects of some antibodies in the panel, the invasive phenotype exhibited proved to be a useful platform for comparing our anti-CDCP1 molecules. The screen was performed in 384-well plates for high throughput analysis, and ultra-high content analysis was used to profile a set of antibodies across different doses, to measure their effects on tissue morphology. Intact 3D image stacks were analysed, allowing spatial information in the z-plane to be retained. Machine learning approaches selected the optimum feature sets for classifying treatment responses. The depth of feature extraction and scale of screening enabled phenotypic clustering to be performed to associate molecules with similar effects on phenotype [33]. Interestingly, this approach successfully clustered the CDCP1-binding antibodies in the screen into two clusters, not one, which had opposite phenotypic effects on PC-3 cells. The high content screen was therefore of value in understanding why the functional screen on primary cells only identified a subset of antibodies later shown to bind to a common antigen, by highlighting differences in their biological effects. We predict that this approach will also enable the clustering of molecules that target different proteins on the same pathway or perturb the same biological process. Potential applications include performing comparisons of the phenotypes induced by novel therapeutics with reference inhibitors of specific signalling pathways to determine mechanisms of action, though profiling of larger well-annotated compound libraries will be needed to evaluate how effectively this can be achieved.

CDCP1 is a type I transmembrane protein with broad expression in normal tissues, but with an established role in cancer progression (reviewed by Uekita and Sakai–[20]). Phosphorylation of its cytoplasmic domain is observed in proliferative cells, and its normal function may involve provision of an anti-apoptotic signal to counteract the loss of cell adhesion contacts during cell division [35]. Proteolytic cleavage of the extracellular domain enhances phosphorylation, which leads to association with Src and PKCδ [32, 36]. Antibodies that prevent this cleavage from occurring have been shown to prevent xenograft growth [22, 32], while cells transfected with cleavage-resistance mutants of CDCP1 are less invasive than cells expressing the wild type protein [32]. However, some uncertainty exists on the link between CDCP1 expression and tumour prognosis, since contradictory effects have been observed in different cancer types [27, 37]. The reason for these differences is not yet fully clear, but recent evidence that links CDCP1 protein expression with oncogenic Ras mutants may help to clarify our understanding of this protein [24].

We identified a group of anti-CDCP1 antibodies in our selection outputs against NSCLC primary cells that were functionally active against the primary cells (and also some established tumour cells lines, Additional file 6: Figure S5B); for example, we observed activation of caspase 3/7 in the primary NSCLC tumour cells when grown as spheroids in the presence of anti-CDCP1 antibodies. After identifying the antigen using cell-surface display of a membrane protein library, we found additional anti-CDCP1 antibodies in our anti-NSCLC selection panel that were not found via the functional screens in the primary cells. Some of these molecules were included in the multiparametric screen using 3-D cultures of PC-3 cells; here, the anti-CDCP1 molecules clustered into two groups that drove opposite phenotypes in the cells. Some molecules acted similarly to αCDCP1-Ab1, which was identified in the original primary cell screens, reducing the invasiveness of the cells and increasing the polarisation of the micro-tumours formed in the 3-D matrix. Other anti-CDCP1 molecules increased invasiveness, showing that antibodies to the same molecular target can have strikingly different effects on the cells. This interesting observation highlights the value of screening primarily for function rather than target specificity. Understanding the mechanism behind this difference in behaviour will require further study. CDCP1 expression has been shown to be induced by expression of constitutively active Ras mutants, while CDCP1 knock-down abrogates Ras-driven invasiveness and migration [24]. PC-3 cells are K-Ras wild-type [38], but they express CDCP1 in a mixture of the full length form and the proteolytically truncated, constitutively active form [36]. One possibility therefore is that the antibodies differ in their modulation of the level of truncated CDCP1 present on the cells.

In contrast with *in vitro *data generated from primary NSCLC cells, our anti-CDCP1 antibody did not inhibit growth of a NSCLC patient-derived xenograft model. However, when our antibody was co-administered with cisplatin, a significant retardation of xenograft growth was observed beyond that caused by cisplatin alone. The lack of xenograft growth inhibition upon anti-CDCP1 therapy as a single-agent could result from several factors, one of which is the individual sensitivity of different tumour models; the *in vitro *screens were performed with cells from a stage I KRas^WT^ tumour, whereas the xenografts were derived from a stage IV KRas^mut^-P53^mut^ tumour that had not been cultured *in vitro *prior to implantation. Unfortunately, the cells used in the xenograft model did not establish well for *in vitro *culture, so it was not possible to directly compare the effect of anti-CDCP1 treatment in both models in the same format. Previous reports have shown anti-CDCP1 treatments with other antibodies can inhibit the growth of xenografts [25], but to our knowledge, all previous data were generated using cell-line xenografts instead of primary cells. Arguably, the primary model used here may therefore be a more representative challenge for assessing an antibody therapeutic, since it may have retained a more disease-relevant phenotype.

Despite the challenging model used in our xenograft experiment, and the lack of efficacy as a single agent, anti-CDCP1 treatment did mediate a significant enhancement of cisplatin efficacy. The mechanism underlying this result requires further investigation, but suggests CDCP1 maybe a promising target for combination therapies. One possibility is that one therapeutic sensitises the cells to the mechanism of the other–for example, anti-CDCP1 therapy may target a mechanism by which the cells adapt to cisplatin toxicity, perhaps related to CDCP1’s role in mediating anoikis-resistance. Another possibility is that the two therapies are effective against different cell populations within the xenografts. Selection for cisplatin resistance in the A2780 ovarian cancer cell line led to decreased DNA hypermethylation around the *CDCP1* gene [39], while DNA methylation near the *CDCP1* gene promoter region negatively correlates with CDCP1 protein levels in breast cancer [40]. A sub-population of cisplatin-insensitive cells in the xenograft, possibly enriched by the donor’s treatment with carboplatin, may therefore be sensitive to anti-CDCP1 therapy. CDCP1 has been identified on cells with phenotypic markers of mesenchymal stem cells or of neural progenitor cells [41], and its expression in pancreatic cancer tissue has been linked to maintenance of cancer stem-cell phenotypes (including gemcitabine resistance) [42].

## Conclusions

This study has described, we believe for the first time, antibody selections performed using primary tumour cells as the source of antigen. The antibodies (including DARPin antibody mimetics) were selected on primary NSCLC cells, then screened against the same cells in three different culture formats. A subset were also screened in a complex 3-D 384-well multiparametric screening assay using PC-3 cells to investigate effects of different treatments upon phenotypes that are not by discriminated by proliferation assays. Among the antibodies and DARPins identified through these screens, we found a group that bound to CDCP1. Closer examination found that these antibodies have distinct effects on cell growth morphologies, that could not be anticipated by knowledge of the antigen alone. Finally, an anti-CDCP1 IgG that had shown functional effects on primary cells in our screens was tested in vivo against a late-stage NSCLC patient derived xenograft; it was shown to have no effect on growth as a monotherapy but caused a significant enhancement of cisplatin efficacy.

## Methods

### Primary NSCLC cells

Fresh frozen primary NSCLC tumors were supplied by Asterand. The material was thawed and cultured in keratinocyte-SFM media (GIBCO 17005-042) containing 2 % heat inactivated FBS in standard 159 cm2 culture flasks. The cells were allowed to attach and actively divide until the flask was roughly 60–70 % confluent. The culture media was completely changed three times a week. To prevent overgrowth of fibroblasts in the heterogeneous culture, the cells were subcultured using the differential trypsinization method provided by Asterand. Several rounds of differential trypsinization provided us with a highly enriched epithelial population. Dividing cells from primary NSCLC cultures were expanded and cryopreserved in liquid nitrogen with medium at various passage numbers up to a maximum of six passages.

### Phage display antibody and DARPin isolation

Phage display cell panning was performed to isolate scFv antibodies and DARPins able to bind to the primary NSCLC cells. For the isolation of scFv antibodies, a naïve human scFv phage display library [43] was used as described previously [44]. DARPins were isolated from a synthetic phage display library containing 1 × 10^9^ unique members. Both libraries were used in cell panning against primary human NSCLC cells, in a similar manner to previously described methods using cell lines [2]. In total, three rounds of scFv cell panning were performed, and two rounds of DARPin cell panning. A total of 1760 individual scFv-presenting colonies were picked from the round 2 and round 3 selection outputs and sequenced by Sanger pyrosequencing, yielding 591 unique sequences after eliminating duplicates. Similarly, 1056 DARPin-presenting phage were obtained, with very high sequence diversity. The unique scFv antibodies and selected DARPins were expressed in *E. coli* culture supernatant and screened for cell binding using a fluorescence-linked immunosorbent assay (FLISA). Antibody binding was detected via a fluorescent secondary antibody to the C-terminal His-tag using a 8200 Cellular Detection System (Applied Biosystems, Carlsbad, CA). For subsequent screening, unique scFv antibodies and DARPins were reformatted as Fc-fusion proteins by sub-cloning into a transient mammalian expression vector, under the control of the CMV promoter, upstream of the human IgG1 Fc domain. The recombinant Fc-fusions were expressed in Human Embryonic Kidney (HEK293) cells and were purified from culture supernatant using PhyTip® columns containing Protein A affinity resin (PhyNexus, Inc, San Jose, CA), according to the manufacturer’s instructions. Prior to target antigen identification and further characterisation, the scFv antibodies were reformatted as standard human IgG1 antibodies, and expressed and purified from Chinese Hamster Ovary (CHO) cells as described previously [2].

### NSCLC tumour cell functional assays

For spheroid culture, cells were grown on tissue culture treated substrate then harvested using 0.05 % trypsin. After neutralising trypsin and pelleting the cells, the cells were resuspended at 10,000 cells per 100 μL in a 0.25 % solution of methocult (StemCell H4100) diluted with filtered culture media. One hundred microlitre of the methocult cell suspension was plated in each well of a non-tissue culture treated round bottom plate (Costar 3788). The cells were gently pelleted then the plates were incubated on a plate shaker for 2 h at 37 °C in a 5 % CO2 incubator. After 24 h, spheroids were treated with antibodies. After 96 h incubation, functional assays were performed to assess anti-proliferative and pro-apoptotic effects, using Cell Titer Glo Luminescent Cell Viability (Promega G7572) and Caspase 3/7 Glo (Promega G8092) assay reagents respectively, according to the manufacturer’s instructions.

For monolayer and low-attachment (anoikis) cultures, cells were grown on tissue culture treated substrate then harvested using 0.05 % trypsin. After neutralising trypsin and pelleting the cells, the cells were resuspended at 10,000 cells per 100 μL in filtered culture media. One hundred microlitre of the cell suspension was plated in each well of a non-tissue culture treated round bottom plate (Costar 3788) for low-attachment cultures, and standard tissue culture-treated flat bottom plates (Thermo 165306) for monolayer. Antibodies were added immediately after plating for the anoikis plates and after 24 h for the monolayer plates. The cells were placed in a 37 °C and 5 % CO2 incubator for 72 h (monolayers) and 96 h (low-attachment), after which time functional assays were performed to assess anti-proliferative and pro-apoptotic effects, using Cell Titer Glo Luminescent Cell Viability (Promega G7572) and Caspase 3/7 Glo (Promega G8092) assay reagents respectively, according to the manufacturer’s instructions.

### Identification of antibody targets

Antibody targets were identified using Retrogenix Cell Microarray Technology, which employs an array of membrane protein cDNAs expressed in HEK293 cells, as described in Turner et al. [26]. Briefly, 2505 expression vectors, each encoding a full-length human cell surface protein, were arrayed across multiple microarray slides. HEK293 cells were grown over the vector array, leading to reverse transfection at each array location. After fixing the cells, the interaction between antibodies and the cells presenting the receptor array was detected using a goat anti-human antibody conjugated to AlexaFluor 647 (Life Technologies, Paisley, UK) and analysed using ImageQuant software (GE Healthcare, Bucks, UK). zsGreen encoded within the library vector was used to define the array positions.

### Transfection of NCI-H358 cells with CDCP1 targeting siRNAs and staining for flow cytometry analysis

To transfect NCI-H358 cells, either Smartpool On-Targetplus CDCP1 siRNA (Thermo/Dharmacon, Catalog # L-010732-00-0005) or On-Targetplus Control siRNA non-Targeting siRNA #1 (Thermo/Dharmacon, Catalog # D-001810-01-05) were combined with Lipofectamine RNAi Max (Life Technologies, Catalog # 13778-150) in Opti-MEM Reduced Serum Medium with GlutaMax Supplement (Life Technologies, Catalog #51985034.) The final concentration of targeting siRNAs & non-targeting siRNAs was 20nM and RNAiMax was used at 1.2 μL per 100 μL reaction. Reagents were mixed gently by pipetting the solution up and down and then incubated for 15 min at room temperature. Fifteen thousand cells/well were plated into a 6-well flat bottom plate containing 100 μL siRNA complex for a final volume of 2000 μL. Cells were incubated at 37 °C, 5 % CO_2_. Three days after the cells were transfected, the cells were harvested using Enzyme Free Cell Dissociation Buffer (Gibco, Catalog # 13151-014) as described in the manufacturer’s dissociation protocol. Cells were re-suspended in FACs buffer (PBS containing 2 % FBS) at 1 × 10^6^ cells/ml. Cells were Fc-blocked with 1 μg of human IgG/10^5^ cells for 15 min at room temperature. After blocking, the NCI-H358 cells were stained for 30 min on ice with either DARPin-Fcs at 1 μg/10^6^ cells; allophycocyanin conjugated anti-CDCP1 antibody (R&D Systems FAB26662A/Lot LVQ0109021X) at 10 μL/10^6^ cells, or an isotype control antibody at 1 μg/10^6^ cells. The DARPins and isotype control antibodies were unconjugated and required secondary antibody staining. The DARPins and isotype control were stained with Alexa Fluor 647 goat anti-human IgG (H + L) (Molecular Probes A-21445) at 10 μg/mL for 45 min on ice. Cells were also stained with propidium iodide to confirm cell viability. After staining, cells were re-suspended in FACs buffer, run on a BD LSRII flow cytometer, and final flow cytometric analysis was performed using TreeStar FlowJo software.

### Recombinant protein expression

DNA sequences encoding the signal peptides and extracellular domains of human CDCP1 [Uniprot:Q9H5V8-1, RefSeq:NP_073753, residues 1-667], its short splice variant [Uniprot:Q9H5V8-3, RefSeq:NP_835488], and mouse CDCP1 [Uniprot:Q5U462-1, RefSeq:NP_598735, residues 1-666] were synthesised (GeneArt) and sub-cloned into a transient mammalian expression vector, under the control of the CMV promoter, upstream of a FLAG-His_10_ affinity tag. The resulting vectors were transfected into HEK293 cells, and the proteins were purified from the culture supernatant by Immobilised Metal Affinity Chromatography (IMAC) using a HisTrap column (GE Healthcare, Bucks, UK). The proteins were eluted with an imidazole gradient, then further purified on a Superdex 75, 16/60 size exclusion column (GE Healthcare, Bucks, UK) pre-equilibrated in PBS.

### CDCP1 ELISA

Enzyme-linked immunosorbent assays (ELISAs) were performed by immobilising 50 μL recombinant CDPC1 per well, typically at 1–5 μg/ml in PBS, on 96-well Maxisorb plates (Nunc) overnight at 4 °C. Bovine serum albumin (Sigma) at the same concentration was added as a negative control antigen to wells on the same plate. The antigen plates were washed three times with PBS and blocked in 3 % non-fat milk powder in PBS, then 50 μL/well antibodies in blocking solution were added and incubated at room temperature for at least 1 h. The plates were washed three times with PBS-Tween, then incubated with 50 μL of appropriate secondary antibody-peroxidase conjugates (goat anti-human-Fc-peroxidase conjugate, Sigma cat # A0170, at 1:10,000 dilution was used for human IgGs and DARpin-Fcs) in 3 % non-fat milk in PBS-Tween for at least 30 min at room temperature, washed again three times in PBS-Tween, and developed for 2–10 min using 50 μL 3,3′,5,5′-Tetramethylbenzidine (TMB) substrate. The reaction was quenched with 50 μL 0.5 M H_2_SO_4_, then the absorbance measured at 450 nm on an Envision microplate reader.

### Matriptase digest of recombinant human CDCP1 in the presence of antibodies

50 nM recombinant human CDCP1 extracellular domain with a C-terminal FLAG-His_10_ tag was treated with 5 nM recombinant matriptase catalytic domain (R + D Systems, cat# 3946-SE) in the presence of 200 nM anti-CDCP1 antibodies or 100 nM aprotinin protease inhibitor. The reaction was incubated at room temperature. Aliquots were taken at specific timepoints, which were quenched in LDS loading buffer (containing reducing agent) and frozen. Samples were analysed by SDS-PAGE and Western blot, probing for the C-terminal FLAG-tag of the recombinant protein.

### Effect of antibodies on CDCP1 levels and cleavage in cell lines by western blot

DU-145, NCI-H358 and HCT116 cells were plated on 6-well plates at 3e5-5e5 cells/well and incubated overnight in media containing 10 % serum at 37 °C/5 % CO_2_. The following morning, the media was aspirated and the cells washed with PBS, then 1 mL media containing 10 μg/mL antibody (αCDCP1-Ab3 from our panel, mouse monoclonal antibody clones 309137 and 309121 (both from R + D Systems), negative control IgG) was added. The cells were incubated with the antibodies for a further 4 h at 37 °C/5 % CO_2_, then washed with PBS, lysed in Triton-X100 and analysed by SDS-PAGE and Western blot, probing for CDCP1 with an antibody to the cytoplasmic C-terminal region (CST #4115).

### PC3 cell 3D tissue culture and image acquisition

PC3 cells (ATCC CRL-1435) were cultured in DMEM/F12 on tissue culture plates with 10 % FBS, detached by trypsinisation, counted and stored in frozen aliquots. Frozen cells were thawed and suspended in InvasogelO-gel-8 (OcellO B.V., The Netherlands), which was selected empirically from ten different gel formulations as supporting the optimum invasive tissue phenotype in 3D culture. Three thousand cells were seeded in 15 μl of gel per well in 384-well plates using a CyBio Selma 96-tip automated liquid handler. Plates were subsequently incubated at 37 °C and 5 % CO_2_ for 30 min. Test DARPin-Fcs, formulated in PBS, or dasatinib in DMSO, were diluted in DMEM/F12 containing 10 % FBS and 45 μl of each diluted antibody was added per assay well. Plates were incubated at 37 °C and 5 % CO2 for 7 days. Each plate was subsequently cooled to 4 °C and 15 μl of cooled fix-stain reagent (OcellO B.V., The Netherlands) was added to each well. Each well was washed twice with PBS and stored at 4 °C.

Plates were imaged using a Pathway-855 automated microscope (Becton Dickinson, Oxford, UK) fitted with a Nikon 4× lens (NA = 0.16) and plates were fed to the imager using a Twister-II plate handler. Two 20-section grayscale image stacks were captured from each well–one fluorescence channel for f-actin staining (EX = 548, EM = 645) and one for nuclei staining (EX = 380, EM = 435). Each section was captured as 1344 × 1024 pixels with 16-bit intensity information.

### Image analysis

Image analysis was performed within OMiner™ (OcellO B.V., The Netherlands). To extract feature data from image stacks, the intensity information in each section of the image stack was scanned and a segmentation mask was generated for each section using WMC segmentation [45]. Objects that were out-of-focus were discarded and the remaining objects from the same channel were aligned based on overlap-ratio. The nuclei were also assigned as children of each organoid based on location.

Phenotypic measurements were extracted per-object per-section. A collection of 70 morphological features were extracted from each channel. Additionally, another 7 correlative features were extracted by comparing relative phenotype between two channels. Object measurements in the same well were further aggregated (mean and standard deviation), resulting in a collection of 294 features. Volume measurements were extracted by aggregating all per-section measurements from the same object. Data for each feature were z-score normalized across the entire experiment, using buffer controls, which were distributed across the assay plates and represented 25 % of the total number of wells.

### Data analysis

Firstly, accumulative phenotypic learning was used to extract a robust feature set that best described the phenotype induced by each antibody at each dose. Feature selection was performed by a pair-wise comparison of the phenotype of the buffer control to each antibody at each dose. For each pair of per-dose DARPin and buffer control measurements, a random forest feature selection [46] was performed to identify 10 features (5 % of all features). Class-wise bootstrap sampling validation using 30 samples was included during random forest feature selection. The cross-validation was repeated 500 times to ensure that the feature selection results were stable. By repeating the feature selection for all pairs of buffer control and DARPin, the frequency estimation of the top-10 features selected from each pairwise comparison was refined to produce a frequency-estimation for all selected features. Finally, the 6 most commonly occurring features were selected that most strongly discriminated treatments based on a 5 % cut-off on the feature frequency estimation. i.e. there will be at least a 5 % chance that any one of these 6 features will be included in the top 10 features when feature selection is performed between any random pair of DARPin and buffer control measurements. This is 16 times higher than a feature being randomly selected from the entire set of 297 features. These features were defined as follows:

#### Invasion inhibition:

A morphological measurement of per-organoid roundness. A perfect spherical organoid will yield the highest [*invasion inhibition*] value while an irregular shape organoid will yield a lower [*invasion inhibition*] value. It is defined by:

$$ \left[ invasion\kern0.28em  inhibition\right]=4\cdot \frac{\left[ per\kern0.28em  organoid\kern0.28em  size\right]}{\pi \cdot {\left[ major\kern0.28em  axis\right]}^2} $$

where [*per organoid size*] is the pixel count of an organoid and [*major axis*] is the maximum distance between any two pixels in this organoid.

#### Total proliferation:

A per-well morphological measurement of the accumulated area of organoid masks over the whole stack. It has an equivalent translation to the total volume of organoids in the 3D image stack. A proliferative cell line will yield a higher [*total proliferation*] value. It is defined by:

$$ \left[ total\; proliferation\right]={\displaystyle \sum_{i=1}^s}{\displaystyle \sum_{j=1}^{n_i}}\left[ Are{a}_{i,j}\right] $$

where [*Area*_*i,j*_] is the size of organoid binary mask of the *j*-th organoid in image section *i. s* is the total section number and *n*_*i*_ is the total organoid count in the image section *i*.

#### Cell polarity:

A per-organoid correlative measurement of the relative position of nucleus in each organoid. It is the shortest projected distance between one nucleus and the organoid boundary line. It measures spatial localization/distribution of nuclei within each organoid in relative to the organoid boundary. An organoid that forms a hollow structure will yield a lower [*cell polarity*] value with low standard deviation σ[*cell polarity*] while a higher [*cell polarity*] value with higher standard deviation σ[*cell polarity*] for a solid structure. It is defined by:

$$ \begin{array}{l}\left[ cell\; polarity\right]=\frac{1}{NC}{\displaystyle \sum_{nc=1}^{NC} \min \left(\left\Vert \left[{M}_{i,nc}\right]-\left[P{B}_{i,j}\right]\right\Vert \right)}\hfill \\ {}\sigma \left(\left[ cell\; polarity\right]\right)=\sigma \left(\left\Vert \left[{M}_{i,nc}\right]-\left[P{B}_{i,j}\right]\right\Vert \right)\hfill \end{array} $$

where *NC* is the number of nuclei in the *j*-th organoid in the *i*-th section, [*M*_*i,nc*_] is the mass center coordinate of one nucleus belonging to the *j*-th organoid in the *i*-th section. [*PB*_*i,j*_] is the collection of all pixels on the boundary line of the *j*-th organoid in the *i*-th section.

#### Organoid branching:

A per-organoid morphological measurement of the average branch length of each organoid. It measures the furthest distance which cells can travel from the main cell cluster of each organoid. The binary mask of the organoid is skeletonized and translated into a graph of edge and vertex in which a branch is defined as an edge with one and only-one single-connected vertex. For each organoid, the length of branches is averaged. It is defined by:

$$ \left[ oraganoid\; branching\right]=\frac{1}{m}{\displaystyle \sum_{b=1}^m\left[{B}_{i,j,b}\right]} $$

Where [*B*_*i,j,b*_] is the *b*-th branch defined in the skeleton of the *j*-th organoid in the *i*-th section. *m* is the total number of branches found in the skeleton.

#### Organoid count:

A per-well morphological measurement of total individual organoid number. It measures how many separated organoids are formed in each well. It is defined by:

$$ \left[ oraganoid\; count\right]={\displaystyle \sum_{i=1}^s{n}_i} $$

where *s* is the total number of sections and *n*_*i*_ is the total organoid count in the *i*-th image section.

#### Per-organoid size:

Per-organoid size is a per-organoid measurement of the per-organoid geometric area in pixels in one section. It measures the growth of each organoid. It is defined by:

$$ \left[ per\; organoid\; size\right]={\displaystyle \sum_x^{M_x}}{\displaystyle \sum_y^{M_y}}{p}_{\left(i,x,y\right)} $$

Where *p*_(*i,x,y*)_ is one pixel in the binary mask of one organoid in the *i*-th section at coordinate (*x*, *y*), and *M*_*x*_ and *M*_*y*_ are the collection of *xy*-coordinates in one organoid.

Secondly, unsupervised phenotypic clustering, using Ward’s method for hierarchical clustering with 2-fold cross-validation [47, 48], was used to group the per-dosage treatment-induced phenotype into different classes, based on the 6 features. Each DARPin-Fc dose was analysed independently and per-well measurements from replicates were aggregated into a single data-entry for noise suppression. The number of clusters (five, designated A to E) was determined empirically based on the Davies–Bouldin index and the Calinski-Harabasz index [49, 50]. These classes are defined by different contributions from the 6 selected features, as shown in Fig.3b and Additional file 5: Table S1. The size of a partition is defined as follows:

$$ \%(i)=\frac{F_i}{\sum_{j=1}^6{F}_j} $$

where *F*_*i*_ is the z-score value of the *i*-th feature, and *F*_*j*_ is the z-score of each of the top 6 features. The values of *F*_i_ and %(*i*) are both signed. Thirdly, a sequence of cluster labels was assigned to antibodies to represent the dose-dependent phenotypic profile. The phenotype at each dose was assigned to a phenotypic class previously defined using unsupervised clustering; this transformed the dose–response into a sequence of class labels.

### Anti-CDCP1 treatment of NSCLC patient-derived xenografts

All procedures were performed in accordance with federal, state and Institutional guidelines and were approved by the MedImmune Institutional Animal Care and Use Committee in an AAALAC-accredited facility. XID (Harlan Laboratories, USA) mice at 4 to 6 weeks of age were implanted subcutaneously with 30 mm^3^ of NSCLC patient-derived tumour fragments which had been previously passaged three times in Rag-2 mice. Tumours were allowed to grow to approximately 100 mm^3^. The animals were then injected intravenously with antibody at 30 mg/kg twice weekly for three weeks and/or with cisplatin at 6 mg/kg every 4 days for three doses. Tumours were measured twice weekly and animals were euthanized when tumours reached 2000 mm^3^.

## Additional files

Additional file 1: Figure S1. Characterisation of NSCLC primary tumours #1 and #2 for in vitro screening assays. Cells from the tumour were cultured in three formats and tested for antibody-mediated growth inhibition (by cell-titer-glo luminescence) and induction of apoptosis pathways (by caspase 3/7-glo luminescence). Tumour #1 cells were sensitive to anti-IGF1R treatment in all settings, which was therefore selected as a positive control in screening assays. Caspase induction by anti-IGF1R was not observed in tumour #1 cells when grown in monolayers at the time point tested, so this assay was not used for screening. Tumour #2 showed sensitivity to anti-EGFR in anchorage-independent and monolayer culture, and to anti-EGFR/anti-IGF1R combination treatment in anchorage-independent and spheroid cultures. (PPTX 314 kb) Additional file 2: Figure S2. (A) Measurement of apoptosis pathway induction upon treating NSCLC tumour #1 cells with the scFv-Fc antibody panel in two culture conditions, measured by Caspase 3/7-Glo luminescence signal. (B) Effects of the DARPin-Fc antibody panel upon NSCLC tumour #1 cell growth in three culture conditions, measured by Cell-Titre Glo (CTG) luminescence signal. All figures are presented as described for in Fig. 2b. (PPTX 650 kb) Additional file 3: Figure S3. (A) GeoMean fluorescence signals for anti-CDCP1 antibodies binding to NCI-H358 cells that were untreated, or treated with non-targetting or CDCP1-targetting siRNA. (B) Binding of anti-CDCP1 antibodies to recombinant full-length CDCP1 transcript variant 1 (Origene cat# TP320633) in a direct ELISA. (PPTX 120 kb) Additional file 4: Figure S4. (A) Anti-CDCP1 antibodies do not protect recombinant CDCP1 extracellular domain (ECD) from cleavage by matriptase catalytic domain, determined by Western blot detection of the C-terminal FLAG tag. (MagicMark XP MW markers were included but were not detected by the anti-FLAG antibody.) (B) Effect of anti-CDCP1 treatment on CDCP1 levels and proteolytic processing in three cell lines, which differ in their intrinsic levels of cleaved/intact CDCP1. Cells were plated on standard tissue culture plates and incubated overnight, then treated with antibodies at 10 μg/ml for 4 h. Triton X-100 cell lysates were probed with an antibody to the CDCP1 cytoplasmic domain (Cell Signalling Technology CST#4115) that detects both intact CDCP1 and the retained fragment of proteolytically cleaved CDCP1. In all three cell lines, antibody αCDCP1-Ab3 from our panel and clone 309121 (R + D Systems MAB26662) caused reductions in the amount of cleaved CDCP1 detected. In NCI-H358 cells and HCT116 cells, the level of intact CDCP1 was also reduced. In contrast, clone 309137 (R + D Systems MAB 2666) had no effect. Clone 309121 (C), but not clone 309137 (D), competes with αCDCP1-Ab3 for binding recombinant CDCP1, measured by direct ELISA on recombinant CDCP1 short isoform. (PPTX 1536 kb) Additional file 5: Table S1. Phenotypic classes assigned in the Multiparametric screen. (DOCX 477 kb) Additional file 6: Figure S5. (A) Morphological changes were observed after αCDCP1-Ab3 treatment of the three cell lines shown in Additional file 3: Figure S3B when grown in anchorage-independent culture. The untreated cells show different growth morphologies in these conditions that correlate with the respective levels of cleaved/intact CDCP1 present (Additional file 4: Figure S4B). DU-145 cells form dispersed small clusters, while HCT116 cells mostly form larger spheroid-like clusters. NCI-H358 cells show intermediate behaviour. (B) Treatment of these cells with αCDCP1-Ab3 in anchorage-independent culture caused dose-dependent decreases in overall proliferation for both DU-145 and NCI-H358 cells, but not HCT116 cells. The treatment also altered the observed growth morphology of the NCI-H358 cells, and to a lesser extent the HCT116 cells, driving the NCI-H358 cells in particular to a more spheroid-like form. (PPTX 410 kb)

## Abbreviations

NSCLC: Non-small cell lung carcinoma
DARPin: Designed ankyrin repeat protein
scFv: Single-chain variable fragment
FMAT: Fluorescent microvolume assay technology
PDX: Patient-derived xenograft
CDCP1: CUB-domain containing protein 1
ELISA: Enzyme-linked immunosorbent assay
PBS: Phosphate-buffered saline
ECD: Extracellular domain
