# Supplementary figures and images for: Identification of anti-tumour biologics using primary tumour models, 3-D phenotypic screening and image-based multi-parametric profiling
Source: Mol Cancer. 2015 Jul 31;14:147. doi: 10.1186/s12943-015-0415-0 (PMC4521473; doi:10.1186/s12943-015-0415-0)

## Slide 1
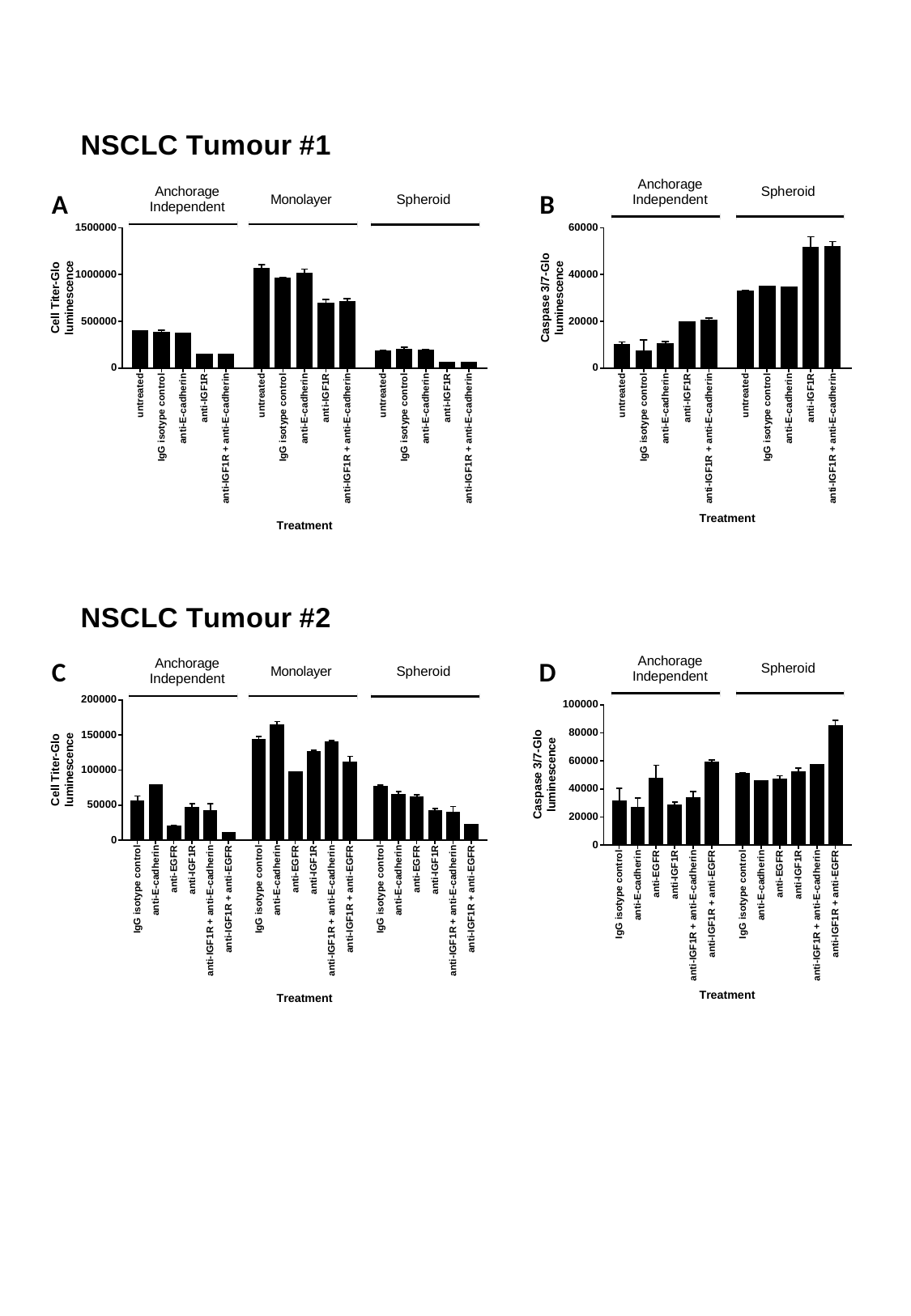

A
B
C
D

Supplement: Additional file 1: Figure S1. — Characterisation of NSCLC primary tumours #1 and #2 for in vitro screening assays. Cells from the tumour were cultured in three formats and tested for antibody-mediated growth inhibition (by cell-titer-glo luminescence) and induction of apoptosis pathways (by caspase 3/7-glo luminescence). Tumour #1 cells were sensitive to anti-IGF1R treatment in all settings, which was therefore selected as a positive control in screening assays. Caspase induction by anti-IGF1R was not observed in tumour #1 cells when grown in monolayers at the time point tested, so this assay was not used for screening. Tumour #2 showed sensitivity to anti-EGFR in anchorage-independent and monolayer culture, and to anti-EGFR/anti-IGF1R combination treatment in anchorage-independent and spheroid cultures. (PPTX 314 kb) [file 12943_2015_415_MOESM1_ESM.pptx]

## Slide 1
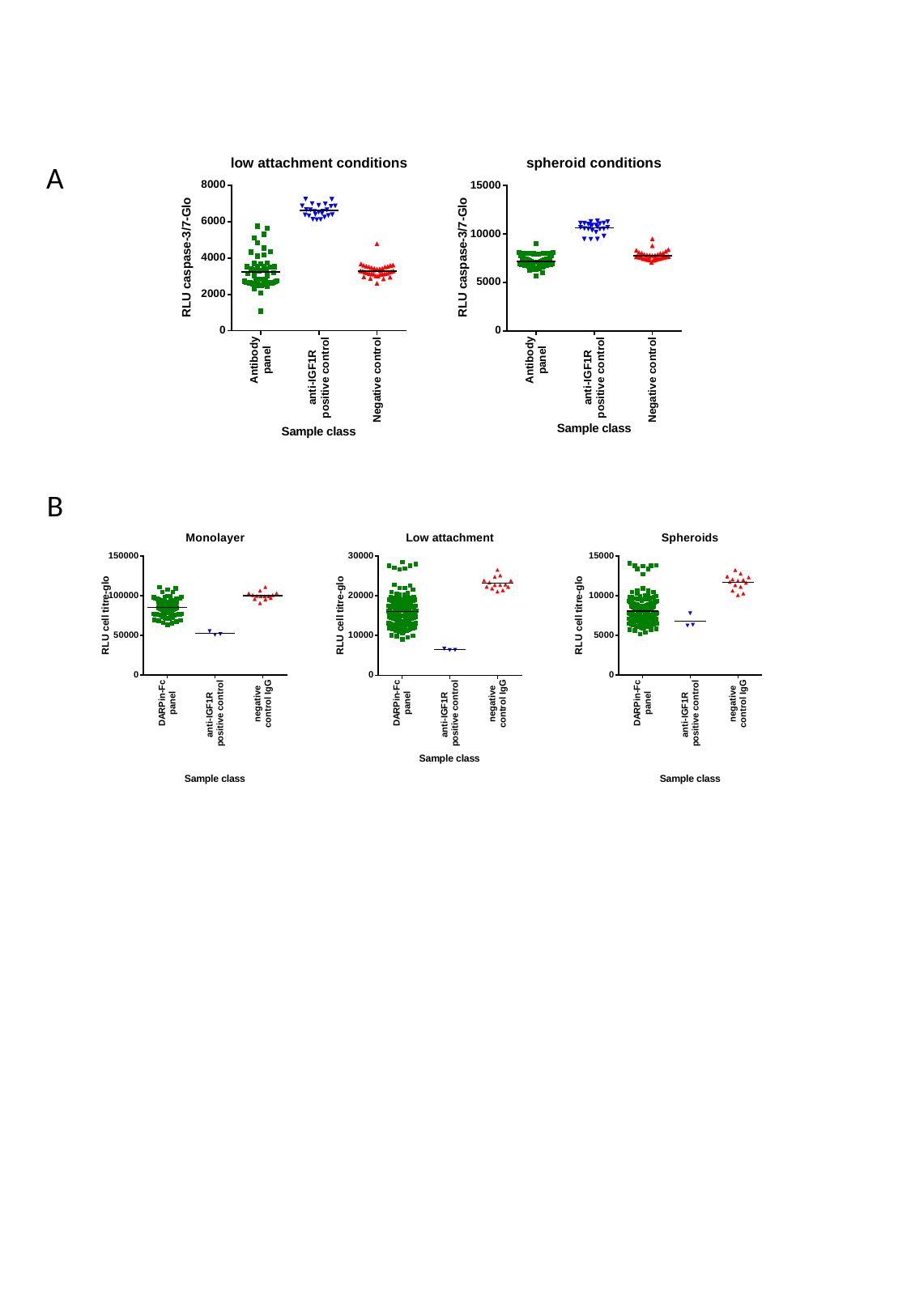

A
B

Supplement: Additional file 2: Figure S2. — (A) Measurement of apoptosis pathway induction upon treating NSCLC tumour #1 cells with the scFv-Fc antibody panel in two culture conditions, measured by Caspase 3/7-Glo luminescence signal. (B) Effects of the DARPin-Fc antibody panel upon NSCLC tumour #1 cell growth in three culture conditions, measured by Cell-Titre Glo (CTG) luminescence signal. All figures are presented as described for in Fig. 2b. (PPTX 650 kb) [file 12943_2015_415_MOESM2_ESM.pptx]

## Slide 1
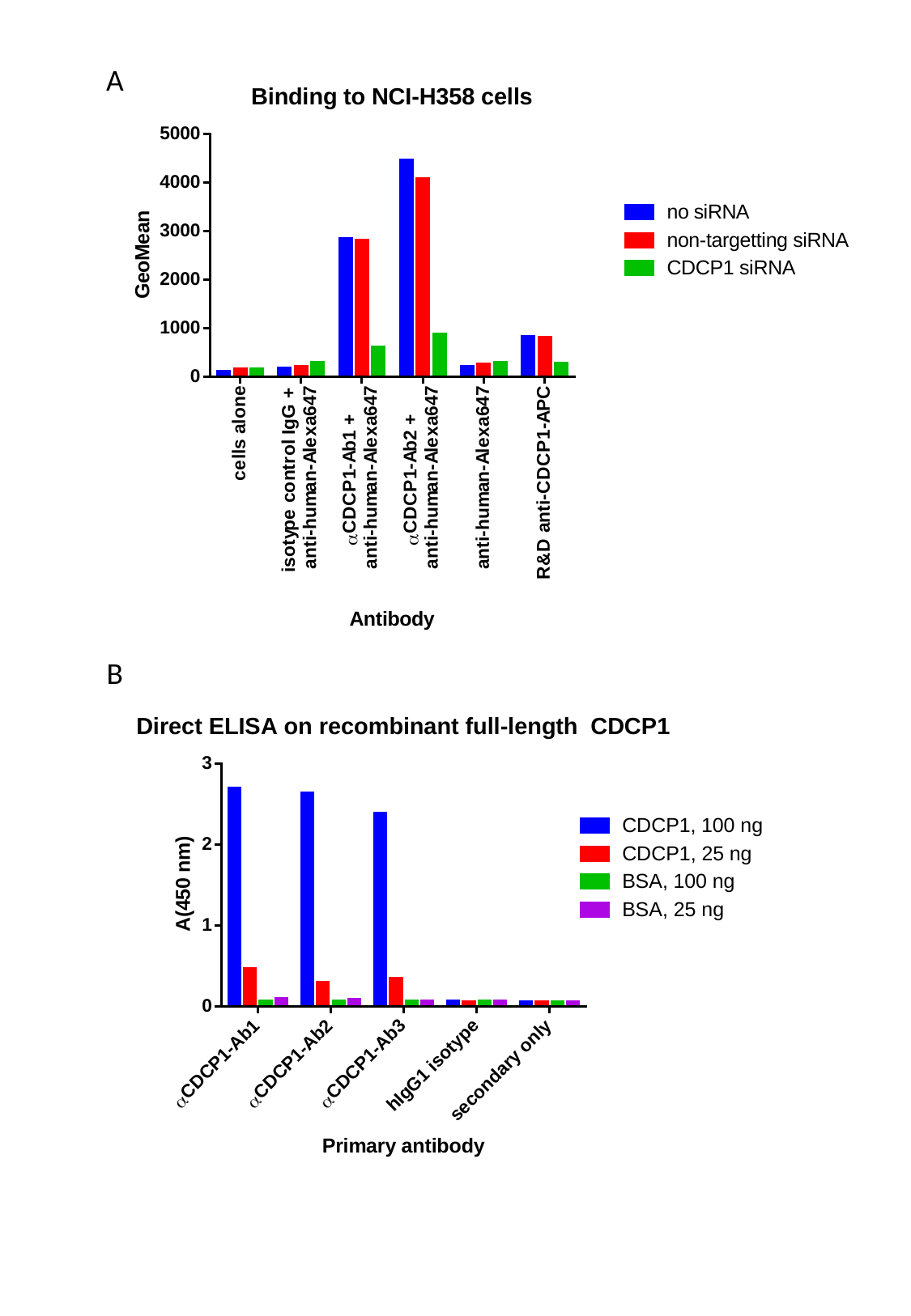

A
B

Supplement: Additional file 3: Figure S3. — (A) GeoMean fluorescence signals for anti-CDCP1 antibodies binding to NCI-H358 cells that were untreated, or treated with non-targetting or CDCP1-targetting siRNA. (B) Binding of anti-CDCP1 antibodies to recombinant full-length CDCP1 transcript variant 1 (Origene cat# TP320633) in a direct ELISA. (PPTX 120 kb) [file 12943_2015_415_MOESM3_ESM.pptx]

## Slide 1
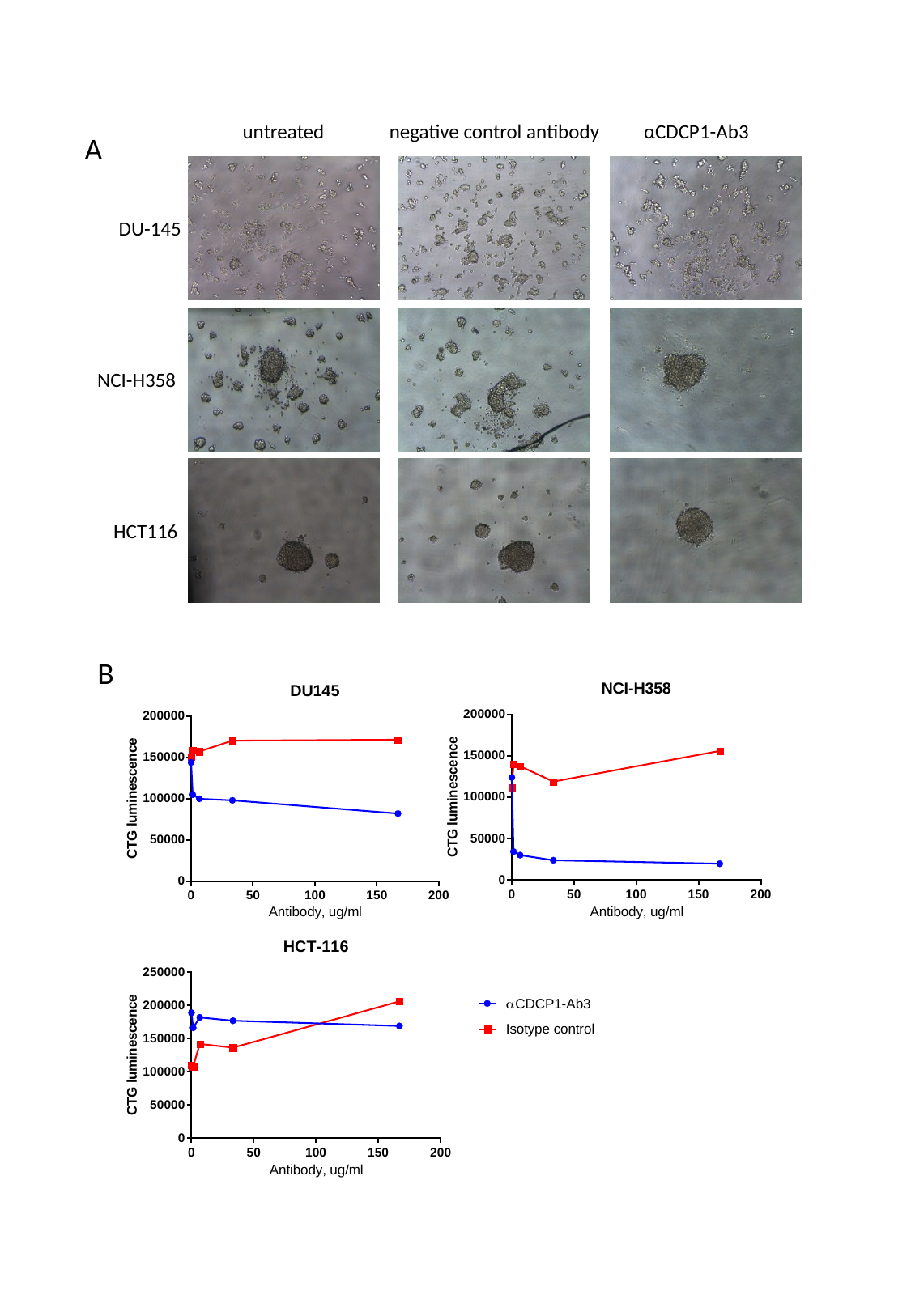

untreated
negative control antibody
αCDCP1-Ab3
DU-145
NCI-H358
HCT116
A
B

Supplement: Additional file 6: Figure S5. — (A) Morphological changes were observed after αCDCP1-Ab3 treatment of the three cell lines shown in Additional file 3: Figure S3B when grown in anchorage-independent culture. The untreated cells show different growth morphologies in these conditions that correlate with the respective levels of cleaved/intact CDCP1 present (Additional file 4: Figure S4B). DU-145 cells form dispersed small clusters, while HCT116 cells mostly form larger spheroid-like clusters. NCI-H358 cells show intermediate behaviour. (B) Treatment of these cells with αCDCP1-Ab3 in anchorage-independent culture caused dose-dependent decreases in overall proliferation for both DU-145 and NCI-H358 cells, but not HCT116 cells. The treatment also altered the observed growth morphology of the NCI-H358 cells, and to a lesser extent the HCT116 cells, driving the NCI-H358 cells in particular to a more spheroid-like form. (PPTX 410 kb) [file 12943_2015_415_MOESM6_ESM.pptx]
