# Supplementary material for: Identification of anti-tumour biologics using primary tumour models, 3-D phenotypic screening and image-based multi-parametric profiling
Source: Mol Cancer. 2015 Jul 31;14:147. doi: 10.1186/s12943-015-0415-0 (PMC4521473; doi:10.1186/s12943-015-0415-0)
Supplement: Additional file 4: Figure S4. — (A) Anti-CDCP1 antibodies do not protect recombinant CDCP1 extracellular domain (ECD) from cleavage by matriptase catalytic domain, determined by Western blot detection of the C-terminal FLAG tag. (MagicMark XP MW markers were included but were not detected by the anti-FLAG antibody.) (B) Effect of anti-CDCP1 treatment on CDCP1 levels and proteolytic processing in three cell lines, which differ in their intrinsic levels of cleaved/intact CDCP1. Cells were plated on standard tissue culture plates and incubated overnight, then treated with antibodies at 10 μg/ml for 4 h. Triton X-100 cell lysates were probed with an antibody to the CDCP1 cytoplasmic domain (Cell Signalling Technology CST#4115) that detects both intact CDCP1 and the retained fragment of proteolytically cleaved CDCP1. In all three cell lines, antibody αCDCP1-Ab3 from our panel and clone 309121 (R + D Systems MAB26662) caused reductions in the amount of cleaved CDCP1 detected. In NCI-H358 cells and HCT116 cells, the level of intact CDCP1 was also reduced. In contrast, clone 309137 (R + D Systems MAB 2666) had no effect. Clone 309121 (C), but not clone 309137 (D), competes with αCDCP1-Ab3 for binding recombinant CDCP1, measured by direct ELISA on recombinant CDCP1 short isoform. (PPTX 1536 kb) [file 12943_2015_415_MOESM4_ESM.pptx]

## Slide 1
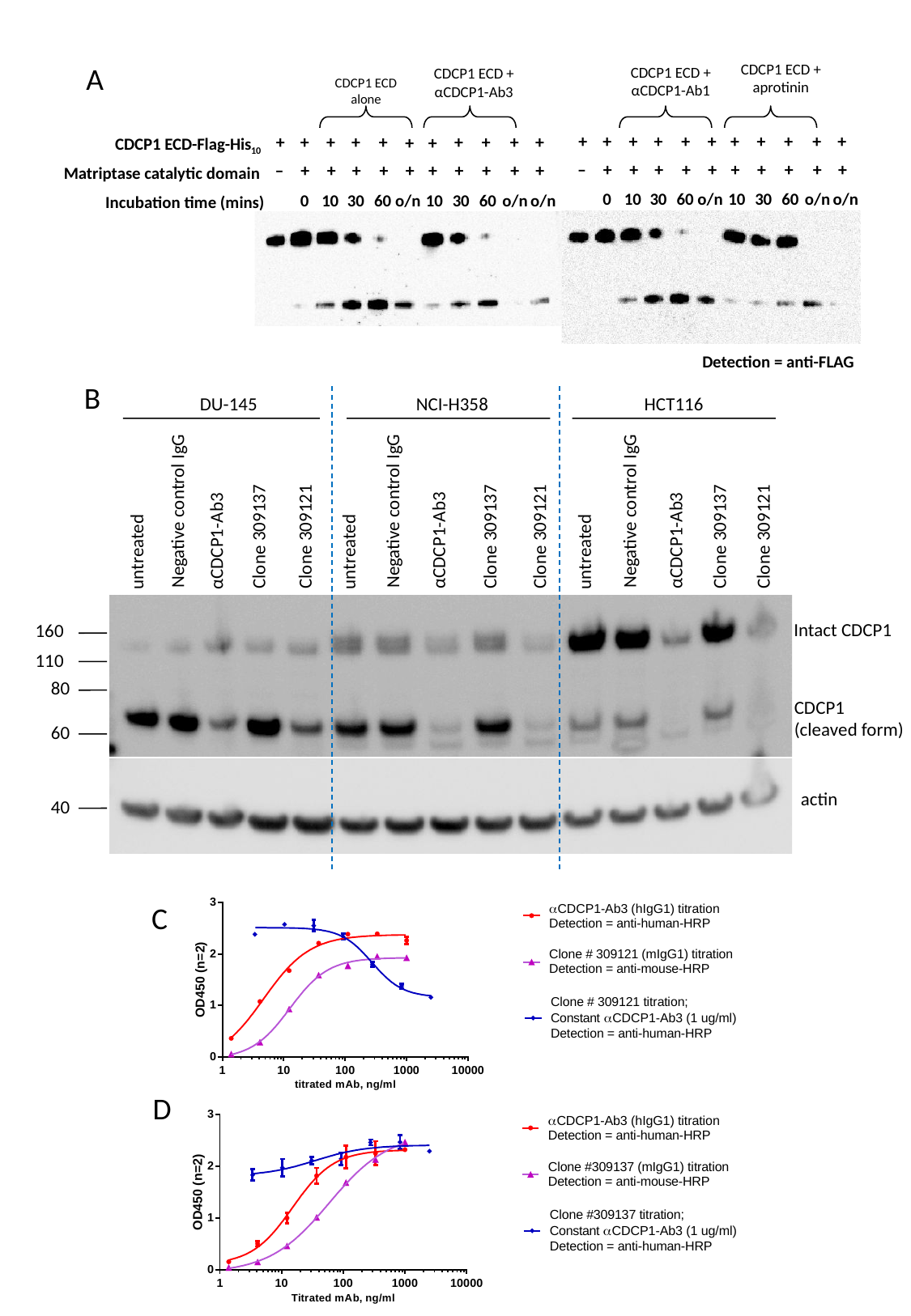

CDCP1 ECD + aprotinin
CDCP1 ECD + αCDCP1-Ab1
CDCP1 ECD + αCDCP1-Ab3
CDCP1 ECD alone
+
+
+
+
+
+
+
+
+
+
+
+
+
+
+
+
+
+
+
+
+
+
CDCP1 ECD-Flag-His10
–
+
+
+
+
+
+
+
+
+
+
–
+
+
+
+
+
+
+
+
+
+
Matriptase catalytic domain
0
10
30
60
o/n
10
30
60
o/n
o/n
0
10
30
60
o/n
10
30
60
o/n
o/n
Incubation time (mins)
Detection = anti-FLAG
A
B
DU-145
NCI-H358
HCT116
Negative control IgG
Clone 309137
Clone 309121
αCDCP1-Ab3
untreated
Negative control IgG
Clone 309137
Clone 309121
αCDCP1-Ab3
untreated
Negative control IgG
Clone 309137
Clone 309121
αCDCP1-Ab3
untreated
Intact CDCP1
160
110
80
CDCP1
(cleaved form)
60
actin
40
C
D
