# Supplementary material for: Identification of anti-tumour biologics using primary tumour models, 3-D phenotypic screening and image-based multi-parametric profiling
Source: Mol Cancer. 2015 Jul 31;14:147. doi: 10.1186/s12943-015-0415-0 (PMC4521473; doi:10.1186/s12943-015-0415-0)
Supplement: Additional file 5: Table S1. — Phenotypic classes assigned in the Multiparametric screen. (DOCX 477 kb) [file 12943_2015_415_MOESM5_ESM.docx]

Table S1: Phenotypic classes assigned in the Multiparametric screen.

| Class | Principle Phenotype  (in comparison with class C) | 3D Surface Reconstruction | Projected 3D Binary Mask |
| --- | --- | --- | --- |
| Class A | Reduced Proliferation, reduced invasion, reduced polarity. Included the dasatinib positive control | 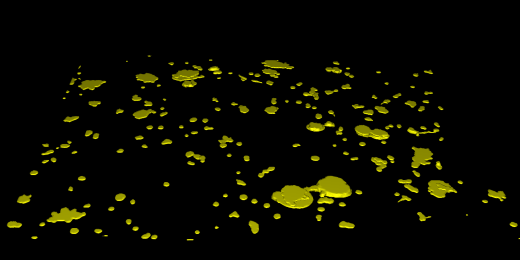 | 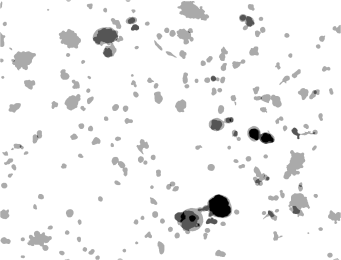 |
| Class B | Reduced invasion, increased polarity.  Typified by a high incidence of organoids lacking protrusions and by the presence of lumens. | 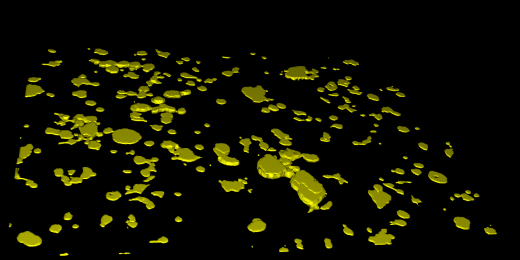 | 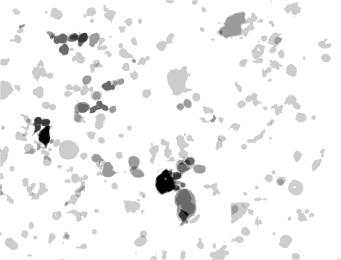 |
| Class C | Phenotype of untreated organoids, including buffer control and non-targeting control antibodies | 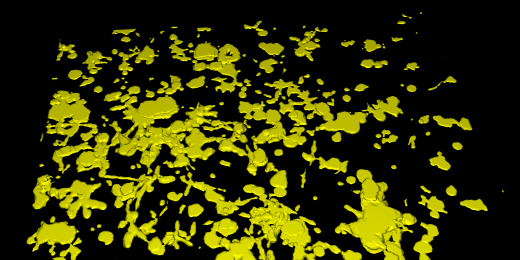 | 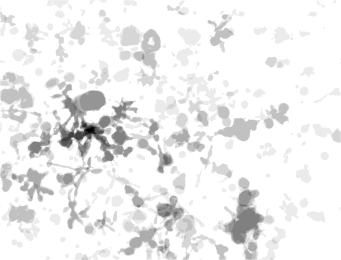 |
| Class D | Increased proliferation and invasion | 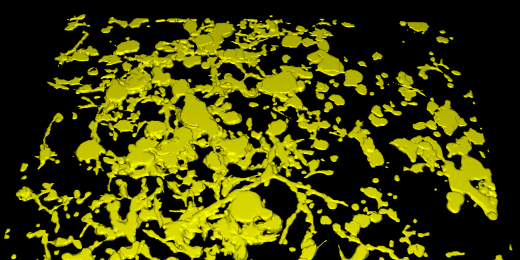 | 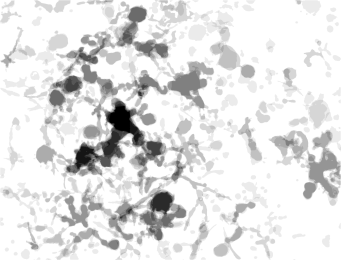 |
| Class E | Increased proliferation and invasion; decreased polarity | 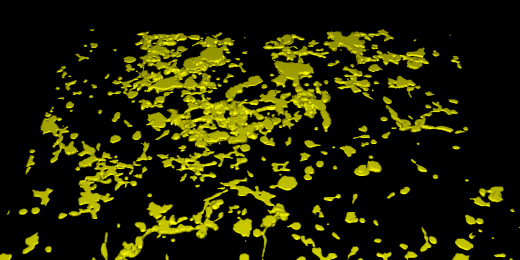 | 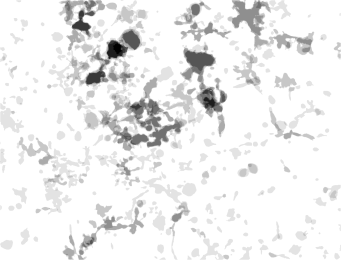 |
